# Peritoneal malignancy in the global COVID-19 pandemic: experience of recovery and restoration in a high-volume centre through NHS and independent sector collaboration

**DOI:** 10.1308/rcsann.2022.0074

**Published:** 2023-12-01

**Authors:** EJ Arbuthnot, J Parker, T Cecil, F Mohamed, R Williams, M Page, B Moran

**Affiliations:** ^1^ Peritoneal Malignancy Institute, Hampshire Hospitals NHS Foundation Trust, UK; ^2^ Hampshire Clinic, UK

**Keywords:** COVID-19, Peritoneal malignancy, Service provision, Independent sector

## Abstract

**Introduction:**

Treatment of peritoneal malignancy with cytoreductive surgery (CRS) and hyperthermic intraperitoneal chemotherapy (HIPEC) requires substantial critical care, theatre and nursing resources. The COVID-19 pandemic caused challenges in providing a high volume, tertiary referral service.

**Methods:**

We reviewed data on referrals and operations performed in a tertiary referral centre in both NHS and independent sector settings. The impact of COVID-19 on activity was assessed using 2019 as a benchmark.

**Results:**

New patient referrals were similar, with 891 in 2019 compared with 833 in 2020. Delivery of CRS and HIPEC operations were initially impacted by COVID-19. NHS and independent sector collaboration facilitated recovery, with 284 patients treated in 2020 compared with 280 in 2019.

**Conclusions:**

Close collaboration and structural organisation between the clinical and management teams in the NHS and independent sectors facilitated recovery and restoration of a complex tertiary referral service for peritoneal malignancy during the COVID pandemic.

## Introduction

Treatment of peritoneal malignancy is a rapidly expanding area of surgical oncology. For most patients peritoneal malignancy is a terminal event, but selected patients can benefit from cytoreductive surgery (CRS) combined with hyperthermic intraperitoneal chemotherapy (HIPEC).^1–5^ CRS combines peritonectomies with resection of involved organs followed by intraoperative HIPEC.^1–5^ The average operating time is 9h, with potential for significant morbidity and occasional mortality.^1–4^ Prolonged disease-free control, and cure, is achievable, particularly in patients with appendiceal tumours,^1,2^ but also in other peritoneal malignancies.^4,6,7^

Commissioning by the English National Specialist Advisory Group (NSCAG) as a national treatment centre for pseudomyxoma peritonei (PMP) of appendiceal origin has allowed the Peritoneal Malignancy Institute (PMI), Basingstoke, to become the highest volume centre globally.^8^ PMP is a rare clinical condition, usually arising from a perforated tumour of the appendix, characterised by mucinous ascites and diffuse peritoneal tumour implants, with figures suggesting an incidence of three to four people per million per year.^9^ CRS and HIPEC is a complex, resource intensive intervention. The long procedures and critical care requirements are challenging in maintaining service delivery, resulting in unprecedented difficulties during the global COVID-19 pandemic. The aim was to outline the experience in a high-volume peritoneal malignancy unit and report successful adaptation mechanisms in the face of this unexpected healthcare crisis.

## Methods

The PMI is a separate clinical business unit with a senior manager and clinical director. PMI is part of Basingstoke General Hospital, a 450-bed hospital with eight level 2 and eight level 3 critical-care beds. Five surgeons focus predominantly on peritoneal malignancy supported by four clinical nurse specialists (CNS). A purpose built 18-bed unit, with 14 single rooms, was opened in 2009 and is adjacent to an eight-bed level 2 high dependency unit. Three rooms have isolation facilities for neutropenic patients. In 2019, up to seven CRS and HIPEC cases were performed per week. Some of these cases were outsourced to the Hampshire Clinic (HC), a local private hospital when demand exceeded capacity. HC has a dedicated intensive care unit with 24h consultant cover provided by the PMI surgeons and anaesthetic teams.

The challenges of delivering a high-volume peritoneal malignancy program during the COVID-19 pandemic is reported, including preparation and reconfiguration, recovery and restoration of services. The 2019 activity was used as a reference to demonstrate the impact on service disruption and recovery after temporarily suspending operative activity. Pandemic responses and strategies are outlined, and early results reported.

## Results

### Pre COVID-19 activity

In 2019, 891 new patient referrals were received at PMI, with 280 having CRS and HIPEC procedures. In 2019, 34/280 (12%) NHS procedures were performed at HC, with an additional 48 privately insured patients. All 328 patients were admitted to level 3 critical care postoperatively for an average of 2 days prior to ward step down.

### Post COVID-19 activity

#### Preparation and reconfiguration

In February 2020, the need for 100 ventilated patients on the Basingstoke site was predicted for COVID-19 pandemic management. All nonurgent surgical interventions were suspended to create critical care capacity and allow preparation time for the influx. Ventilation resources were transferred from HC to the NHS site, ceasing all HC surgery.

The PMI ward, adjacent to the high dependency unit, was converted to a bespoke COVID-19 intensive care facility. Highly skilled PMI nurses and the CNSs were deployed to frontline services in critical care and COVID-19 wards. The remaining peritoneal malignancy patients were cared for by general surgery nurses.

Management and communication with large numbers of PMI outpatients was transferred to administration staff and surgeons. Outpatient appointments were converted to telephone or video consultations. For patients awaiting operations, the responsible consultant informed them of the need to defer surgery. Oncology teams were contacted to consider resuming, or continuing, chemotherapy for appropriate patients.

#### Recovery

An NHS England contract with the independent sector was implemented and PMI worked with HC to explore opportunities for reinstating service provision. Major issues included safety to patients and staff from COVID-19, service relocation to green areas and green pathways through theatres, critical care and the ward.

**Figure 1:**
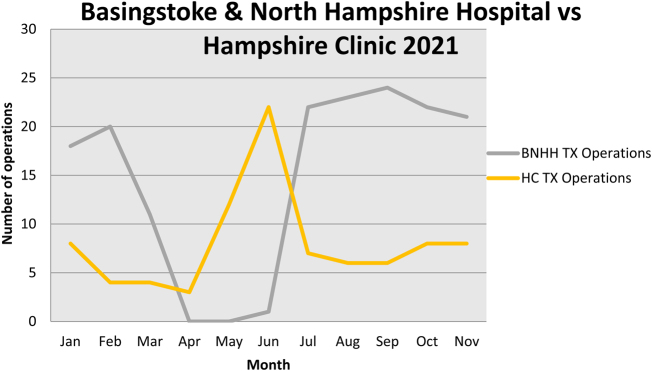
CRS and HIPEC procedures in Basingstoke (grey line) and HC (yellow line). The cessation of treatment in Basingstoke was accommodated by a surge in HC until recovery in July. CRS = cytoreductive surgery; HC = Hampshire Clinic; HIPEC = hyperthermic intraperitoneal chemotherapy

CRS and HIPEC cases initially continued at HC until the ITU ventilators were moved to meet demand on the Basingstoke site. No cases were performed on the Basingstoke site in April or May 2020 and only one in June. PMI relocated all CRS and HIPEC to HC, with a peak of 22 cases in June. This was supported by redeployment of PMI nursing and medical staff. The trend in patients undergoing CRS and HIPEC at the two sites is demonstrated in Figure 1.

A waiting list of 94 patients accumulated prior to recovery plans. A small number of urgent life-threatening patients were treated after detailed negotiation with other providers. Two were in the Wellington Hospital, London, operated on by PMI surgeons in conjunction with Wellington consultants and PMI anaesthetists. Another from Northern Ireland was treated in PMI Dublin.

In June 2020, CRS was recommenced in Basingstoke. Across both sites, there were 23 and 29 cases in June and July, respectively. Patients were accommodated on the private ward at the Basingstoke hospital site with green pathways and single rooms for COVID-safe care. This allowed admission of CRS and HIPEC patients while the original PMI ward continued as a COVID-19 ward. All patients admitted for treatment were screened for COVID-19 by nasal swabs. A computed tomography (CT) scan of the chest, abdomen and pelvis on admission for surgery was performed, looking for radiological evidence of COVID-19 and significant disease progression to inoperability.

#### Restoration

Similar to the 891 referrals in 2019, there were 833 new referrals to PMI in 2020. There were 324 operations performed, with more NHS cases (284 versus 280) and fewer private operations (36 versus 48) compared with 2019. In 2020, 32% (92/284) of NHS cases were performed at HC compared with 12% in 2019. There was one postoperative death during the pandemic from abdominal sepsis. One patient contracted COVID-19 as an inpatient at the beginning of the pandemic. A further two patients tested positive in 2021 on day 3 (community acquired) and on day 7 (possibly nosocomial spread). All were detected on routine PCR swab testing and recovered fully with no adverse effects.

## Discussion

CRS and HIPEC is a complex strategy for selected patients with peritoneal malignancy with 5-year survival in excess of 60%, particularly patients with appendiceal malignancy.^1–3^ The safe and effective delivery of a high volume, complex tertiary referral unit is challenging in a crisis such as the COVID-19 pandemic. Issues revolved around competing for high value resources, particularly critical care and senior nursing capacity. Benefits were provided to the hospital for COVID-related admissions due to the skills and resources previously in place for this high-volume peritoneal malignancy service. The release of highly skilled PMI nursing staff for the treatment of COVID-19 patients was also invaluable, although many developed COVID-19 with acute and long-term consequences for the staff and the service recovery.

All CRS and HIPEC units have substantial critical care requirements. Service cessation before the predicted peak allowed preparation time in providing critical care beds, anaesthetic and nursing staff, with Basingstoke being able to free more capacity as compared with similar sized hospitals for the increased critical care requirements. Agile managerial, clinical and nursing negotiation with effective organisation secured a timely recovery. This involved relocating the service to HC, with its historical facilities, resources and experience in CRS and HIPEC. This included a pre-existing contract for treating NHS patients, an established consultant on-call rota and a governance framework.

With hindsight and reflection, there are things we might have done differently. The removal of the ventilation facilities from HC to the main NHS hospital, following a national policy, impeded ongoing service delivery and delayed recovery. Predictions of ITU capacity had suggested that all ventilators and staff would be required on the main site. Thankfully the numbers predicted did not materialise.

The skill set of ward-based and CNS nursing staff is a critical resource in peritoneal malignancy. As happened in Basingstoke, these skilled staff are often the first to be called on in a crisis. CNS nurse redeployment impacted significantly on the need for communication with a large number of outpatients. Many patients felt they were at risk of dying from COVID-19 due to immunosuppression or being labelled as high risk, such as those who had splenectomy. Patients awaiting surgery or review felt terrified on two fronts, with fear of COVID-19 infection competing with their concerns of disease progression and uncertainty as to when PMI could resume outpatients or surgical interventions. The administration team and surgeons took on much of this communication. This was not as effective as the CNSs who have established experience, knowledge and skills in this complex area of care and support for the patient journey.

Our experience suggests that disease-specific CNS roles should be maintained in crisis situations, if possible, to maximise remote communication to a highly vulnerable patient population. The use of the dedicated PMI ward was key to the response to the pandemic. Nurses caring for patients outside of their usual skills impacted greatly on psychological wellbeing and created issues in rebuilding a nursing team for service recovery.

CT imaging of all patients before surgery helped to ensure they were less likely to have occult COVID-19 but was also a valuable check for disease progression. Three patients had significant progression of colorectal peritoneal metastases and arguably had been spared an intervention not in their best interests in this unplanned trial of time. They, and their carers, may feel that opportunity for curative treatment was lost, which was, and will be, a concern for many patients with cancer during the pandemic.

Lessons from this experience may help in forthcoming COVID-19 challenges and any future crisis. A recognition of the benefits, threats and challenges to a high-volume surgical service is called for to maximise resources and minimise collateral damage to people with potentially curable advanced cancer. Safe delivery of complex surgical interventions requires flexibility, audit and evaluation to ensure adequate resources are maintained.

## Conclusion

A responsive and agile clinical management structure will allow for anticipation of future pandemic surges to create capacity. It is equally important to plan and adapt for urgent recovery and restoration to mitigate harm to patients with malignancy. The outcomes reported demonstrate the benefits of open collaboration and cooperation between healthcare providers.
